# Optical coherence tomography angiography (OCTA) of retinal vasculature in patients with post fever retinitis: a qualitative and quantitative analysis

**DOI:** 10.1038/s41598-021-96715-8

**Published:** 2021-09-03

**Authors:** Srinivasan Sanjay, Santosh Gopi Krishna Gadde, Sameeksha Agrawal, Padmamalini Mahendradas, Nivedhitha Govindaswamy, Ankush Kawali, Chaitra Jayadev, Sajjan Sangai, Abhijit Sinha Roy, Rohit Shetty

**Affiliations:** 1 Department of Uveitis and Ocular Immunology, Narayana Nethralaya, 121/C, Chord Road, Bangalore, Karnataka 560010 India; 2 Department of Retina, Narayana Nethralaya, 121/C, Chord Road, Bangalore, Karnataka 560010 India; 3 Imaging Bio Mechanics and Mathematical Solutions Lab, Narayana Nethralaya Foundation, #258/A Hosur Road, Bommasandra, Bangalore, Karnataka 560099 India; 4 Department of Cornea and Refractive Surgery, Narayana Nethralaya, 121/C. Chord Road, Bangalore, Karnataka 560010 India

**Keywords:** Immunological disorders, Infectious diseases, Biomedical engineering

## Abstract

Post fever retinitis is a heterogenous entity that is seen 2–4 weeks after a systemic febrile illness in an immunocompetent individual. It may occur following bacterial, viruses, or protozoal infection. Optical coherence angiography (OCTA) is a newer non-invasive modality that is an alternative to fundus fluorescein angiography to image the retinal microvasculature. We hereby describe the vascular changes during the acute phase of post fever retinitis on OCTA. Imaging on OCTA was done for all patients with post fever retinitis at presentation with 3 × 3 mm and 8 × 8 mm scans centred on the macula and corresponding enface optical coherence tomography (OCT) scans obtained. A qualitative and quantitative analysis was done for all images. 46 eyes of 33 patients were included in the study. Salient features noted were changes in the superficial (SCP) and deep capillary plexus (DCP) with capillary rarefaction and irregularity of larger vessels in the SCP. The DCP had more capillary rarefaction when compared to the SCP. The foveal avascular zone (FAZ) was altered with an irregular perifoveal network. Our series of post fever retinitis describes the salient vascular features on OCTA. Although the presumed aetiology was different in all our patients, they developed similar changes on OCTA. While OCTA is not useful if there is gross macular oedema, the altered FAZ can be indicative of macular ischemia.

## Introduction

Post fever retinitis is seen 2–4 weeks after a systemic febrile illness caused by either bacteria, viruses, or protozoa, in an immunocompetent individual^1^. Uveo-retinal manifestations include solitary or multifocal patches of retinitis, localised or generalised involvement of the retinal vessels in the form of beading of the vessel wall, tortuosity, and perivascular sheathing, and macular serous detachment or oedema, and optic nerve involvement^1,2^. Optical coherence tomography angiography (OCTA) is a recent advancement which is a non-invasive alternative to fundus fluorescein angiography (FFA) and indocyanine green angiography (ICGA) to assess the retinal and choroidal microvasculature^3,4^. Dye-based angiography has been found to be useful in assessing retinal vascular involvement and in detecting occlusive complications in retinitis^5^. Extensive leakage of the dye may obscure adequate visualisation of microvasculature in eyes with retinitis. Compared with FFA and Indocyanine green angiography (ICGA), which are the current retinal/choroidal angiographic gold standards, OCTA acquires volumetric scans that can be segmented to specific depths, uses motion contrast instead of intravenous dye, can be obtained within seconds, provides accurate size and localization information and delineates both the retinal and choroidal vasculature. Disadvantages are its limited field of view, inability to demonstrate leakage, increased potential for artifacts (blinks, movement, vessel ghosting), and inability to detect blood flow below the slowest detectable flow^6–11^.

Standard enhanced depth imaging (EDI) optical coherence tomography (OCT) is only capable of showing the structure of choroid and choriocapillaris. However, the OCTA, being rapid, non-invasive and repeatable, is useful for the assessment of the foveal avascular zone (FAZ) and microvascular changes along with segmental imaging and evaluation of the superficial capillary plexus (SCP) and deep capillary plexus (DCP) in several retinal vascular diseases^6–11^. FAZ and capillary density can be measured at both the SCP and DCP^12^. Despite the lack of standardised protocols for image acquisition and interpretation of image scans, OCTA is widely used for the detection of pathophysiology, early diagnosis, treatment and determination of the progression in patients, especially with vascular pathology^4^. It provides good delineation of the pathology along with volumetric data with the ability to show both structural and blood flow information^5^. It can therefore be vital in understanding the vascular changes in eyes with post fever retinitis.

## Methods

This prospective, observational, cross-sectional study with protocol title “FAVOUR”: “Fever Associated Visual Outcome in Uvea and Retina” was approved by the Narayana Nethralaya Hospital Ethics Committee (EC Ref No: C/2018/08/05).

The research followed the tenets of the declaration of Helsinki and an informed written consent was obtained from all study subjects.

The study included 46 eyes of 33 patients who presented with post fever retinitis between August 2018 and July 2020. All patients underwent imaging at presentation to our tertiary eye center on a spectral domain (SD) OCTA system (ANGIOVUE, OPTOVUE, Inc., Fremont, CA, USA) using the Split Spectrum Amplitude Decorrelation Angiography (SSADA) algorithm to quantify vasculature structure, the FAZ, and the superficial and deep retinal vascular plexus densities, by a single trained operator. Scan areas of 3 × 3 mm and 8 × 8 mm centred on the fovea for imaging both superficial and deep retinal plexus were obtained separately for each patient similar to what we have described earlier^13^. The OCT scanner has a scan speed of 70,000 A-scans per second with 304 A scans per B-scan and 304 B-scans per volume. The axial and transverse resolution of the device is 5 µm and 15 µm, respectively. If the signal strength index was less than 40, the scans were repeated and those with poor signal strength were not included.

Tropicamide 1% eye drops was used for dilatation of pupil in all patients. A total of 5 scans were acquired for every patient using an internal fixation target. Only the best quality scans were chosen for analyses. Exclusion criteria for OCTA images were those with motion artefacts, double vessels or undue stretching of vessels. Foveal centre for OCTA images was correlated with OCT scans at the same levels. A parafoveal ring of 1–2.5 mm diameter, proportional to the total area was used for analysis relative to the density of vascular flow.

Non flow mode in SCP and DCP was used to measure the FAZ. ANGIOANALYTICS (OPTOVUE, Inc., Fremont, CA, USA) blood vessel measurement was used to measure automated vessel flow density. Segmentation was performed both by automated and manual techniques, especially in case of gross oedema or poor scan features.

Local fractal dimension was used to represent the presence of vessels in OCTA scans^13^. Calculation of the ratio of local fractal dimension of each pixel in an OCTA image to the maximum fractal dimension was done as described in earlier^13^. Coloured contour, normalised ratio to provide a pictorial representation of an apparent probability index of the presence of vessel was done. Visual comparison of the normalized ratio map with the OCTA image was used to develop a scoring system. The vessel density was computed as a percentage, by counting all the pixels with a normalized ratio between 0.7 and 1.0 and then dividing by the total number of pixels in the OCTA image^13^. Capillary dropouts is a significant parameter to distinguish between normal and diseased eyes. In this study, capillary dropouts were labeled as "spacing between large vessels" and "spacing between small vessels". Spacing between or around the large vessels with a normalized ratio between 0.0 and 0.3 were considered. Pixels in regions around closely packed small vessels, which may be branching out from a large vessel or surrounding small vessels, with a normalized ratio between 0.3 and 0.7 were termed as “spacing between small vessels”^13^.

Gadde et al. have described local fractal dimension to calculate vessel density and FAZ area in a normal healthy Asian Indian population^13^. Quantification of vascular parameters can be affected by projection artefacts (PAs) in the DCP. Govindaswamy et al.^14^ have described the methodology to reduce the PAs.

The inbuilt software from the instrument allows the users to choose flow and non-flow area on a selected layer. OCTA indices from local fractal analysis differentiate the large and small vessel regions of the non-flow area^13^. Thus we used custom OCTA metrics rather than using inbuilt software of the machine for the quantitative analysis.

Deep capillary plexus was found to be the affected initially in most of the retinal vascular disorders. We have previously reported that a significant vascular loss in different grades of diabetic retinopathy at the level of deep vascular plexus after removal of projection artifacts. Before the removal of projection artefacts, this was not apparent with the presence of projection artefacts^2^. Our approach is software-based where a normalized cross-correlation between superficial and deep layer was estimated as a scaling factor to subtract the projection artifact. Hence, it can be presumed that irrespective of the instrument used to obtain and the algorithm used to re-construct angio images, the software-based approach would provide comparable results.

### Conference presentation

Oral presentation at a meeting: Preliminary data at 35th Singapore-Malaysia Joint Meeting in Ophthalmology in conjunction with the 1st Asia–Pacific Ocular Imaging Society Meeting, held from 17 to 19 January 2020 at the Academia, Singapore General Hospital Campus.

### Informed consent

Written Informed consent was obtained from all study subjects.

## Results

Thirty-three consecutive patients with post fever retinitis (46 eyes, 13 bilateral) in the age 18–73 years (median age 40 years) who underwent OCTA between August 2018 and July 2020 were included in the study (Male:Female—17:16). The presumed etiologies for the post fever retinitis were dengue, rickettsia and typhoid fever based on the serology as illustrated in Table 1. The predominant clinical picture was of multifocal retinitis with macular oedema.

**Table 1:** Shows demographic, aetiological and descriptive characteristics in patients with post fever retinitis.

Serial.no	Age	Sex	Eye	Serology	OCT	CMT (in µ)	OCTA
1	28	M	LE	WFT	SRF + middle retinal band like HR along OPL	361	Capillary rarefaction in DCP > SCP Gross vessel dropouts in DCP FAZ enlarged on DCP Darker areas peripapillary corresponding to the disc edema in all the layers, more pronounced in OR and CC Radial lines/streaks along PMB apparent on the CC layer Coarse architecture of CC layer maintained otherwise
2	20	F	RE	Dengue IgG WFT	Middle retinal HR + spongy edema	216	SCP grossly normal DCP shows darker areas corresponding to the retinal edema superiorly along ST arcade FAZ maintained Diffuse changes in coarse architecture of CC
			LE		Inner and middle retinal edema with IRF and SRF	1230	Gross capillary rarefaction in SCP and DCP Enlarged and broken FAZ- vessel loss more appreciable along PMB OR has areas of shadowing and dark patches CC layer shows a better extent of the involved area with radial streaks of alternating darker and lighter areas
3	41	F	LE	WFT	LE IRF + gross SRF with HRS	618	Capillary rarefaction beyond macular area, more prominent in DCP- large patches of darker areas corresponding to the areas of IRF FAZ enlarged and altered in SCP and not seen totally in DCP Darker areas more prominent again in OR and CC layers PMB and ST arcade involved more
4	39	M	RE	WFT	SRF + inner and middle retinal edema- HRS middle retina	371	Extensive capillary rarefaction DCP > > SCP FAZ landmarks obliterated Radial streaks seen in OR and CC layer- noted on even on enface OCT
			LE		SRF + inner and middle retinal edema- HR middle retina and SRHRM	325	SCP -uniform capillary rarefaction, only larger vessels seen FAZ altered and enlarged in SCP No vascular landmarks were visible in DCP No FAZ seen in DCP Radial streaks noted in DCP and more prominent in CC Shadowing of areas in OR
5	49	F	RE	Dengue IgG WFT	↓ CFT OR atrophy	143	Enlarged FAZ- due to CFT Patchy capillary rarefaction both in SCP and DCP Prominent choroidal vasculature in OR and CC
			LE		LE minimal spongy edema PRL and RPE alterations at fovea	230	Patchy areas of rarefaction in SCP > DCP Enlarged FAZ in both SCP and DCP Darker areas in OR corresponding altered architecture in CC layer
6	29	M	LE	WFT	SRF + middle retinal HE SRHRM more inferiorly	468	Inferior capillary rarefaction beyond perimacular area Enlarged FAZ on DCP Artefacts on OR Prominent radial streaks inferiorly with wider bulb like dilatations-cystoid spaces in the CC layer
7	23	F	RE	WFT	SRF + middle retinal and SR HRS	200	SCP normal DCP radial streaks seen nasally continuing into OR and more prominent in the CC Prominent vasculature on DCP FAZ normal
8			LE	WIDAL	SR scar + taut posterior hyaloids face + traction + inner and middle retinal thickening	402	Prominent perimacular network in DCP Grossly normal FAZ Change in coarse architecture in CC OR Normal
9	34	M	RE	WIDAL	RE SRF + SR HRM -middle retinal HR	415	Prominent perimacular vasculature in DCP > SCP Enlarged FAZ in DCP NV complex nasally in OR and CC Coarse architecture in CC
10			LE	WFT	SRF + HR middle retina and SRHRM	373	Rarefaction at DCP Increased FAZ, shadow on OR corresponding to edema Radial streaks in OR and more prominent in CC
11	26	M	RE	WIDAL	SRF with SRHRM	393	Grossly Normal SCP DCP showed capillary rarefaction nasally FAZ normal Prominent perimacular vasculature on DCP
			LE		SRF with SRHRM	481	Grossly normal SCP DCP shows capillary rarefaction nasally more in LE Radial streaks seen in CC layer FAZ normal Prominent perimacular vasculature on DCP
12	24	M	LE	Dengue IgG	SRF + inner and middle retinal thickening + HRS	763	Images poor quality- complete distortion of vasculature, CNP OR and CC not clearly visualised
13	59	F	RE	WFT	SRF + HRS middle retina, inner thickening	495	Gross rarefaction in SCP and DCP
			LE		Minimal SRF and spongy thickening	312	Patchy areas of rarefaction SCP and DCP
14	18	M	RE	WIDAL	SRF – HR SR	322	Prominent perimacular vasculature DCP OR and CC altered texture
			LE		Inner and middle retinal thickening	217	Rarefaction more in SCP > DCP Distorted and enlarged FAZ Shadowing in DCP, OR and CC
15	56	M	RE	Dengue IgG	SRF + IRF Inner retinal thickening	707	Gross capillary rarefaction DCP >> SCP Distorted and enlarged FAZ- artefacts seen inferiorly Brighter shadows inferiorly in CC layer
			LE		SRF + IRF Inner retinal thickening	657	Gross capillary rarefaction DCP > > SCP more inferiorly Enlarged and distorted FAZ Shadows of IRF on OR and CC
16	60	M	RE	WFT	SRF + IRF—inner and middle retinal thickening	915	Rarefaction in DCP > > SCP FAZ maintained in SCP, enlarged in DCP Irregular shadows and artefacts In OR and CC Shadows of IRF and SRF on OR and CC
17	68	M	LE	Dengue IgG	SRF + middle retinal HRS	649	Rarefaction in both SCP and DCP FAZ normal in SCP and DCP
18	18	F	RE	WFT	Intraretinal HRS	831	Normal SCP Generalised and non specific rarefaction at DCP, more inferonasally Shadows of IRF and SRF on OR and CC
19	73	M	LE	Negative	SRF + IRF, inner retinal thickening along PMB	297	Generalised capillary rarefaction in both SCP and DCP Darker areas corresponding to IRF on all the layers
20	18	F	RE	Negative	SRF + inner retinal thickening-middle retinal HR-SR precipitates	233	Generalised extra macular capillary rarefaction in DCP > SCP Enlarged FAZ on DCP Brighter areas on OR And CC layers Darker areas inferiorly corresponding to SRF
			LE		SRF + inner retinal thickening-middle retinal HRS SRHRM	370	Generalised extra macular capillary rarefaction in DCP > SCP Enlarged FAZ on DCP Brighter areas on OR And CC layers Darker areas inferiorly corresponding to SRF
21	23	F	RE	Dengue IgG	Middle retinal thickening	258	Minimal capillary rarefaction in DCP FAZ normal in SCP and DCP
			LE		SRF + inner and middle retinal thickening	690	Gross capillary rarefaction SCP and DCP Distorted FAZ DCP > SCP Darker areas in DCP, OR and CC- corresponding to intraretinal edema
22	26	M	RE	Dengue Ig G	WNL	215	Capillary rarefaction temporal to the disc both at SCP and DCP Rest of macula normal
			LE		WNL	196	Capillary rarefaction temporal to the disc both at SCP and DCP Rest of macula normal
23	20	M	RE	Serology Negative	SRF + middle retinal HR SRHRM	496	Capillary rarefaction DCP > SCP Enlarged FAZ in DCP > SCP Shadowing in OR and diffuse radial streaks in CC layer
			LE		SRF + middle retinal HR SRHRM	486	Rarefaction in SCP > DCP Shadowing in OR Radial streaks in CC layer
24	55	F	RE	Serology Negative	SRF + middle retinal HR	385	SCP is normal DCP shows diffuse non specific capillary rarefaction with artefacts of striations with enlarged FAZ CC has prominent striations with brighter areas corresponding to the middle retinal HR
25	73	F	LE	WFT	Inner and middle retinal thickening nasal to the fovea along PMB	233	Capillary rarefaction both in SCP and DCP—more nasally Enlarged FAZ in both layers Shadowing in OR and CC layers
26	21	M	RE	Dengue	Focal PRL loss	221	Focal rarefaction in DCP
27	53	F	RE	Dengue IgM—Positive	RE cilioretinal A occlusion	228	RE gross CNP in SCP and DCP- poor fixation
			LE			212	Non specific rarefaction in SCP and DCP
28	38	M	RE	WFT	SRF + inner retinal thickening	249	Rarefaction in DCP > SCP Enlarged FAZ DCP > SCP
29	56	M	LE	Serology negative	↓ CFT- middle retinal HE SRHRM PRL disruption	413	Patchy rarefaction in SCP and DCP Altered FAZ Change in CC architecture
30	23	F	LE	Dengue IgG	HRM- middle and OR HR SRF, SRHRM	426	Rarefaction DCP Gross shadowing in OR and CC and DCP FAZ minimally altered
31	40	M	LE	Dengue IgG	↓ CFT	229	CNPs inferiorly with breach in FAZ inferiorly in DCP Corresponding shadowing in OR
32	42	F	LE	Dengue IgG	SRF + IRF + Middle retinal HRS	532	Rarefaction in DCP > SCP Altered FAZ Shadowing in DCP, OR, CC
33	35	F	RE	Dengue IgG	SRF + inner retinal thick + HR + large SRHRM	618	Rarefaction in DCP > SCP Altered FAZ Shadowing in DCP, OR, CC Radial striations in CC
			LE		SRF + inner and mddle retinal thickening + HRS + SRHRM	642	Rarefaction in SCP/DCP over PMB Shadowing in OR and CC Radial striations in CC

*M* Male, *F* Female, *RE* Right eye, *LE* Left eye, *WFT* Weil Felix Test, *OCT* Optical Coherence Tomography, *CMT* Central macular thickness, *HR* Hyper-reflective, *HRS* Hyper-reflective spots, *SRHRM* Subretinal Hyper-reflective material, *CFT* Central Foveal thickness, *SCP* Superficial capillary plexus, *DCP* Deep capillary plexus, *FAZ* Foveal avascular zone, *SRF* Subretinal fluid, *IRF* Intraretinal fluid, *HE* Hard exudates, *CC* Choriocapillaries, *OR* Outer retina, *PMB* Papillomacular bundle, *PRL* Photoreceptor loss, *OPL* Outer plexiform layer, *CNP* Capillary non-perfusion, *ST* Superotemporal, *SR* Subretinal

### Quantitative analysis

**Figure 1:**
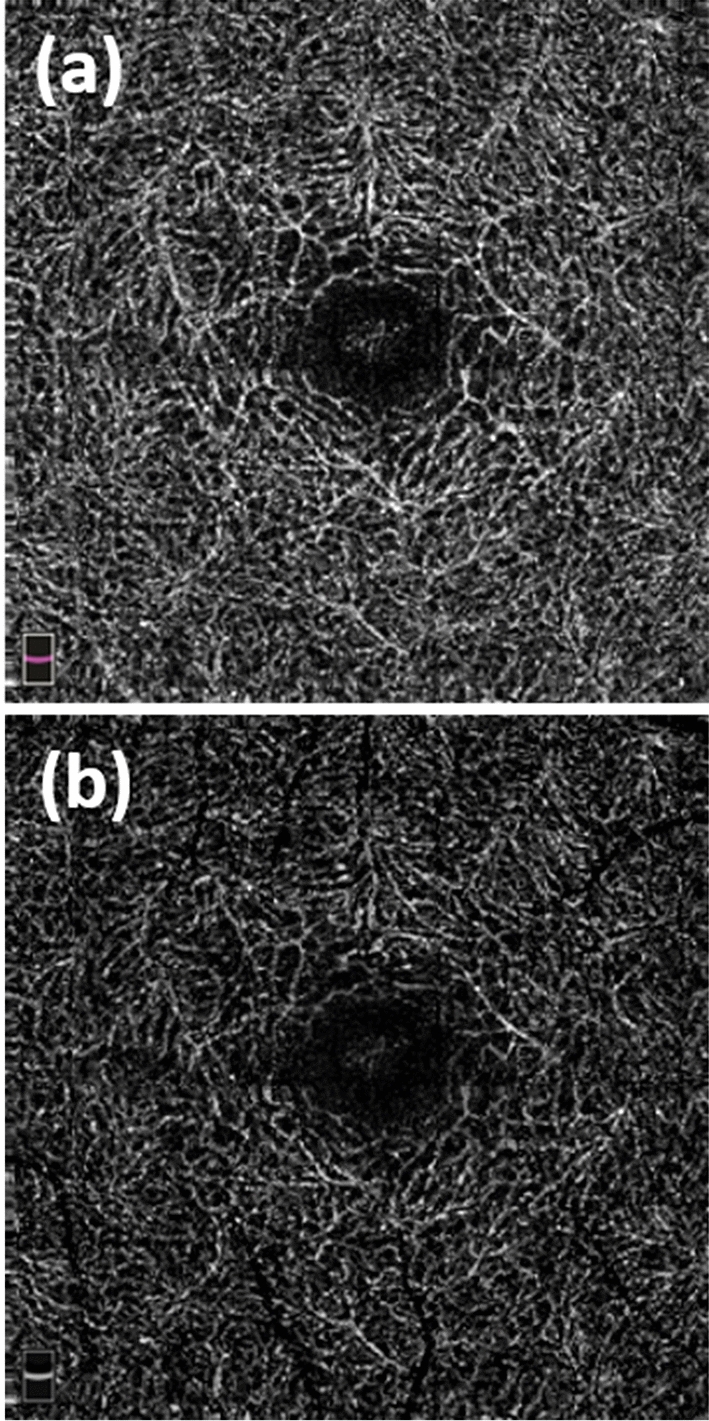
OCTA image of deep layer (3 × 3 mm). (**a**) Original image exported from OptoVue. (**b**) Deep layer after removal of projection artefacts from the superficial layer. *OCTA* Optical coherence tomography angiography.

Figure 1 shows the pre and post removal of projection artefact (PAs). In the SCP, the large vessel spacing was increased with small vessel spacing similar to a normative data. The DCP was more affected in terms of small and large vessel spacing and the vessel density was also significantly lower.

**Figure 2:**
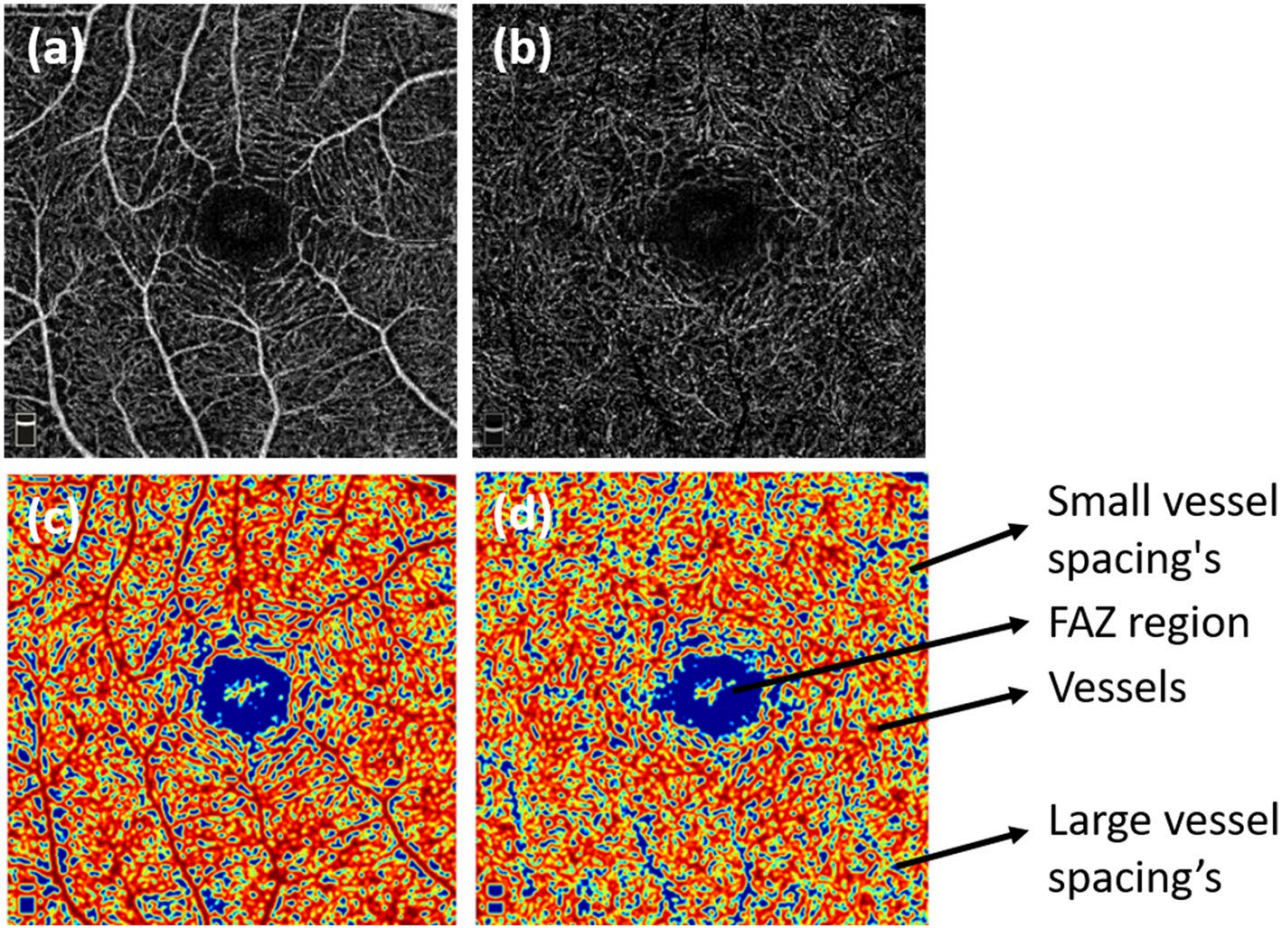
Optical coherence tomography angiography (OCTA) image of superficial (**a**) and deep layer after removal of projection artifacts (**b**). (**c**, **d**) are the respective images after fractal analysis. Regions in red pixels correspond to vessels, blue corresponds to large vessel spacing, and yellow corresponds to small vessel spacing.

The FAZ was not affected during the acute stage in both the SCP and DCP. Figure 2 shows the fractal analysis after removal of the artefacts. The vascular parameters of small and large vessels in the superficial capillary and deep capillary layers are shown in Table 2.

**Table 2:** Showing the vascular variable parameters of small and large vessels in superficial capillary and deep capillary layers of our series against age matched normal.

Vascular parameters	Normative data^13,14^	Post fever retinitis median with 2 SD included	95% CI
FAZ area (superficial)	0.42 ± 0.01	0.431	0.393–0.502
Small vessels spacings (superficial)	36.97 ± 0.32	40.190	39.078–41.403
Large vessels spacings (superficial)	14.85 ± 0.46	25.718	23.239–29.503
Vessel density (superficial)	48.17 ± 0.69	31.436	29.382–35.085
FAZ area (deep)	0.42 ± 0.01	0.407	0.363–0.452
Small vessels SPACINGS (DEEP)	34.03 ± 0.39	42.744	42.170–43.381
Large vessels spacings (deep)	12.19 ± 0.33	24.623	21.024–26.866
Vessel density (deep)	53.77 ± 0.64	32.284	29.715–35.004

Normal age group: 20–67 years.

Our series: 18–72 years.

### Qualitative analysis

**Figure 3:**
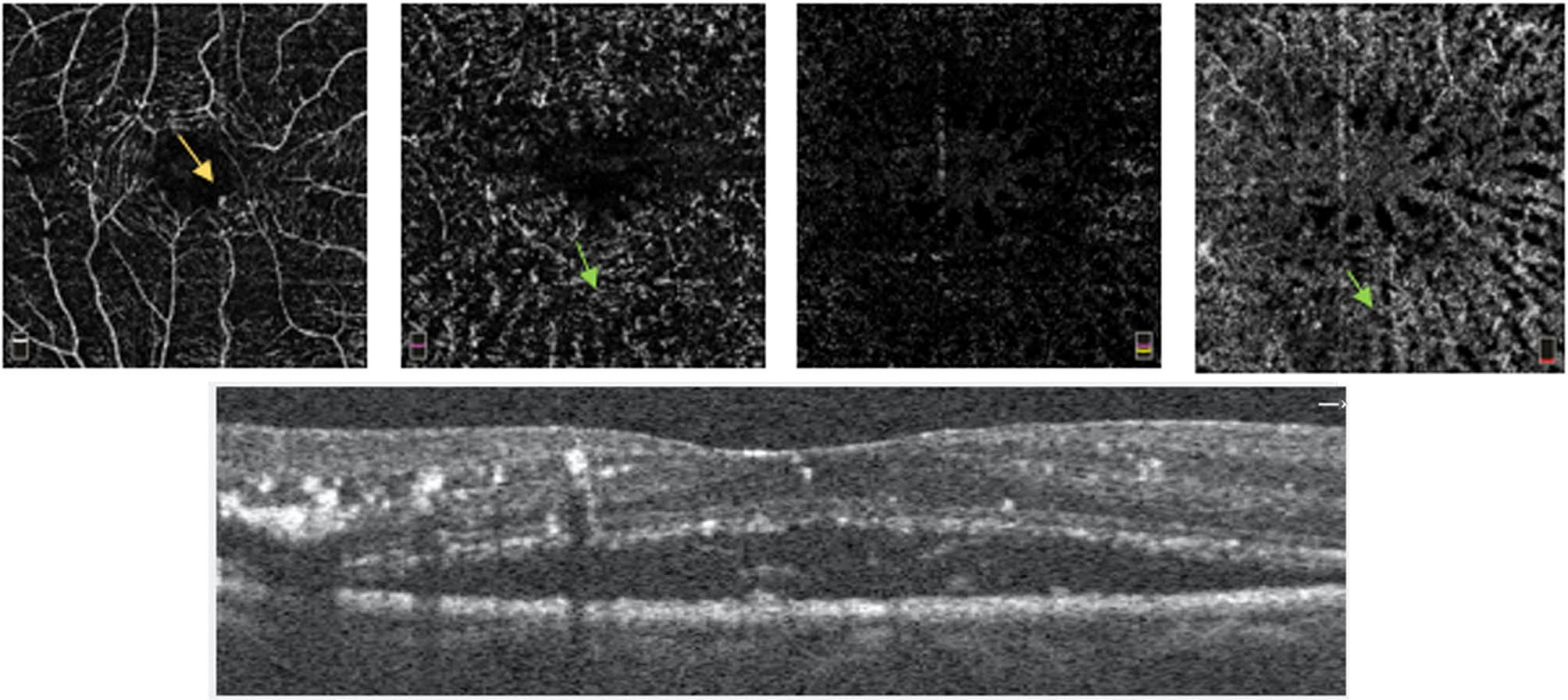
(Patient 24) OCTA: 3 mm × 3 mm section. SCP—Capillary rarefaction seen between larger vessels. FAZ maintained. Beading and aneurysmal dilatation of perifoveal vasculature (yellow arrow). DCP—Diffuse capillary rarefaction seen as radial darker stripes (green arrow) corresponding to retinal oedema in Henle’s layer. Areas of pruning noted in the smaller network of vessels. OR—Projection artifacts with darker radial lines continuing in the avascular layer. CC—Loss of the regular coarse architecture. A better appreciation of the darker radial striations corresponding to oedema in the Henle’s layer (green arrow). OCT showing mild intraretinal oedema and HRS and SRF with subretinal hyperreflectivity. *OCTA* Optical coherence tomography angiography. *SCP* Superficial capillary plexus, *DCP* Deep capillary plexus, *OR* Outer retina, *CC* Choriocapillaries, *OCT* Optical coherence tomography, *FAZ* Foveal avascular zone, *HRS* Hyperreflective spots.

**Figure 4:**
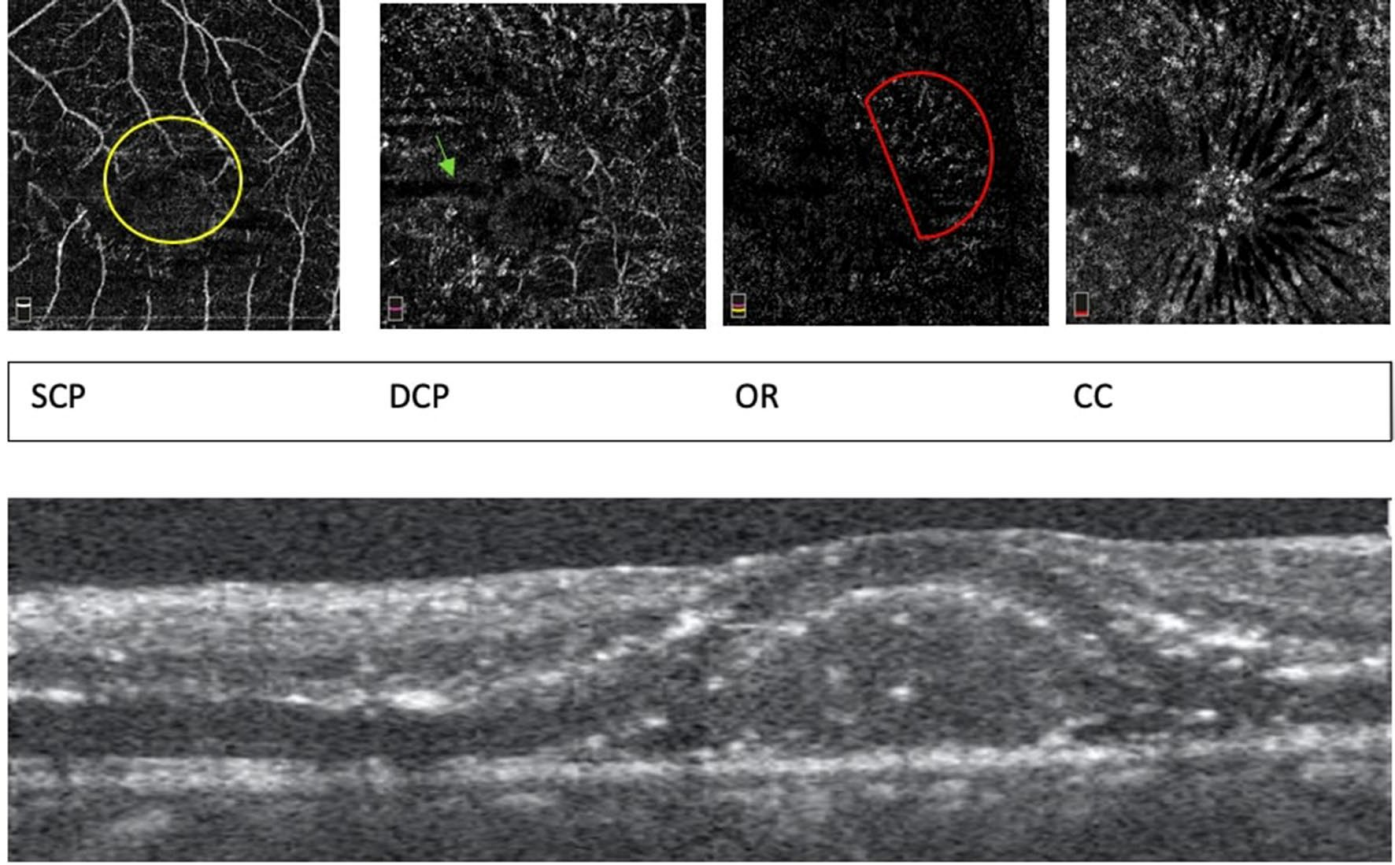
(Patient 33) OCTA: 3 mm × 3 mm scans of SCP, DCP, OR and CC layers. SCP: Extensive capillary rarefaction in between larger vessels and gross distortion of the FAZ landmarks owing to the significant oedema. (yellow circle). DCP: Projection artefacts with poor demarcation of finer vascular network and a totally lost FAZ due to intraretinal oedema (green arrow). Avascular areas of FAZ showing HR brighter areas due to suspending particle artifacts. OR: Few areas of brighter areas due to the overlying projection artifacts. (red D). CC: Changes in the coarse architecture. Radial striations well delineated due to the oedema in Henle’s layer. OCT: Active retinitis with extensive intra retinal oedema, HRS in all the retinal layers and subretinal hyper reflective material. *OCTA* Optical coherence tomography angiography, *SCP* Superficial capillary plexus, *DCP* Deep capillary plexus, *OR* Outer retina, *CC* Choriocapillaries, *OCT* Optical coherence tomography, *FAZ* Foveal avascular zone, *HRS* Hyperreflective spots.

**Figure 5:**
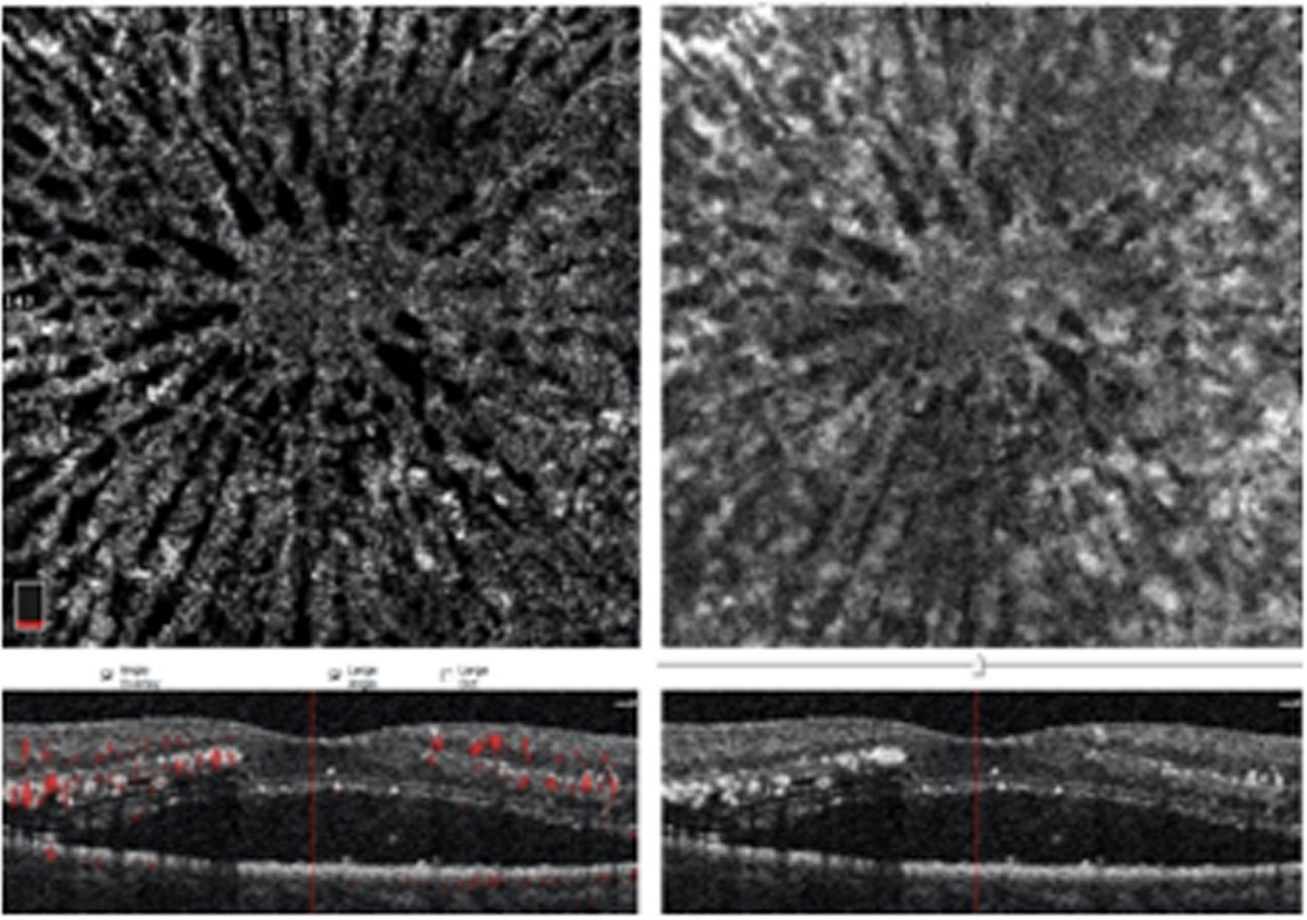
(Patient 24) A 3 mm × 3 mm scan showing CC layer (left image) with Enface projection (right image) of active retinitis. A well demarcated alternating bands of dark and lighter radial striations corresponding to areas of retinal edema in Henle’s layer. *CC* Choriocapillaris.

**Figure 6:**
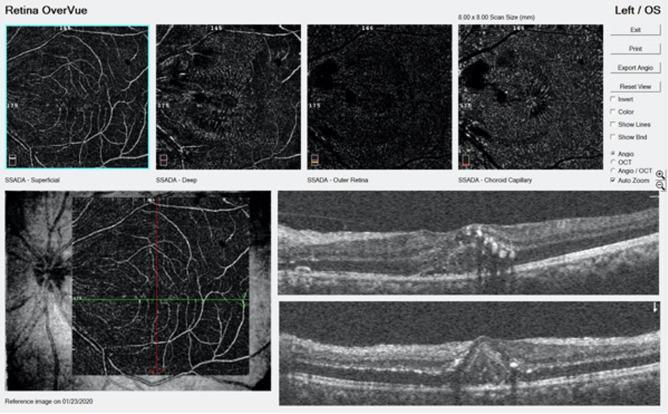
(Patient 24) OCTA 8 mm × 8 mm scan: Larger scan sections show the extent of retinal involvement in active retinitis. Larger vessels in SCP can be seen. Finer vasculature in SCP and DCP are poorly appreciated due to lesser resolution in larger scans. The macular involvement can be estimated from DCP onwards. A CC section shows the radial striations with brighter signals corresponding to the intraretinal HRS. A corresponding OCT B scan shows the severity and retinal involvement. *OCTA* Optical coherence tomography angiography, *SCP* Superficial capillary plexus, *DCP* Deep capillary plexus, *OR* Outer retina, *CC* Choriocapillaries, *OCT* Optical coherence tomography, *FAZ* Foveal avascular zone, *HRS* Hyperreflective spots.

Salient features of active retinitis on OCTA included changes in both SCP and DCP with capillary rarefaction and irregularity of larger vessels in SCP. Pruning of the vessels was noted in SCP and DCP. The FAZ was altered with a broken perifoveal network suggestive of macular ischaemia on 3 × 3 mm scans in patients with active retinitis and areas of retinal thinning on OCT. Capillary rarefaction was better appreciated in the DCP than SCP. Bright hyper-reflective material on OCT was seen corresponding to the areas of capillary rarefaction and artefacts at DCP. The choriocapillaris (CC) layer had a loss of the normal coarse architecture corresponding to areas of retinal oedema on OCT and on the enface image. Both intra retinal and sub retinal oedema had a shadowing effect on the DCP, outer retina (OR) and CC layer causing artefacts thus impeding an accurate measurement of the vascular density and confirmation of the dropout areas. Middle retinal thickening, highly reflective spots (HRS) and hard exudates were seen as bright areas in the DCP and CC and in the OR in some scans. These areas of HRS with middle retinal oedema causing radial striations in the Henle’s layer were more apparent in the CC layer, which may reverse with disease resolution. Inner retinal thickening and middle retinal thickening corresponded with capillary rarefaction in the SCP and DCP, respectively. FAZ enlargement could not be accurately documented in all patients as many presented with macular edema during the acute phase. Figures 3 and 4 shows an OCTA 3X3mm scan in mild and severe intra retinal edema, respectively. Figure 5 shows CC layer with enface projection of active retinitis. Figure 6 shows an 8X8 mm scan which is useful to detect the extent of retinitis, but has poorer vascular resolution compared to 3X3 scans.

## Discussion

OCTA has the ability to non-invasively provide details of retinal and choroidal vasculature, which helps us better understand the microvascular changes in eyes with retinitis, which cannot be delineated well on FFA due to the vascular leakage in inflammatory conditions.^11^ Few studies in post fever retinitis have described changes in the SCP/DCP following dengue and chikungunya retinitis^15–17^. Agarwal et al. reported that there were flow void areas in the SCP, DCP and choriocapillaris slabs in chikungunya retinitis^14^. Aggarwal et al. have also described the OCTA features in acute macular neuro-retinopathy post dengue fever showing disruption of both the SCP and DCP, flow deficit in the foveal region and an increase in the FAZ. They propose that the presence of hairpin loop configuration of the adjacent retinal capillaries is suggestive of retinal capillary ischaemia, which persisted even at their last follow up^16^. Bajgai et al.^17^ noted a broken FAZ in only the SCP of OCTA in a case of dengue maculopathy and these changes persisted at follow‑up visits. Kahloun et al.^18^ described hypointense areas in the SCP/DCP but larger hypointense areas in the DCP/OR/CC on their swept source (SS) OCTA in a patient with rickettsial retinitis. On follow up six weeks later the hypointense greyish areas of retinal capillary nonperfusion persisted in both SCP/DCP^18^.

Khatri et al.^19,20^ have described microaneurysms (MA) on OCTA based on their signals. MAs were classified as high flow MA s and low flow MAs. High Flow MAs show a signal on OCTA. Due to pericyte loss however these high flow MAs are prone to thickening of macula due to leakage and are often are located in the DCP. Nearly three quarters of them were located adjoining cystoid spaces.

Low Flow MAs do not appear on OCTA, they may however be visible on fundus photo or other enhanced techniques.

Schreur et al.^21^ in their study of retinal MA in patients with diabetic macular edema (DME) by OCTA found that MA with focal leakage and located in a thickened retinal area were more likely to be detected on OCTA. In their study MAs were located in intermediate and deep plexus.

In our series of post fever retinitis, microvascular abnormalities were noted in the SCP and DCP with quantifiable changes in both the smaller and larger vessels. Capillary rarefaction areas corresponding to retinitis patches and pruning of vessels was seen in the active phase. The DCP showed profound capillary rarefaction when compared to the SCP due to the involvement of the middle retinal layers. Our series did not show any individual MAs on OCTA. The CC slabs showed signal void areas which can be attributed to shadowing caused by the overlying retinitis patch similar that reported by Shanmugam et al.^22^ The regular vascular pattern or the “angio-architecture” in SCP and DCP was lost in active retinitis, the intraretinal edema and exudation causing an impression of vessel drop out. The flow void areas in the choriocapillaris layer are due to the shadow effect of the superficial edema on to the choroid resulting in loss of the regular coarse architecture. These changes are reversible in non-ischemic retinas once the active inflammation subsides.

In a patient with post typhoid fever neuroretinitis, OCTA showed macular thickening and neuro sensory detachment. Choroidal imaging showed abnormal “patchy” flow voids in the choriocapillaris-likely suggestive of a sluggish blood flow or ischemia. Deep range imaging (DRI) of the choroid revealed increased choroidal thickness and dilated choroidal vasculature, indicating a concurrent choroidal inflammation^23^.

In our series choroidal imaging had artifacts in acute stages due to intraretinal fluid. In cases where choroidal imaging was possible, we noted altered choroidal architecture with darker areas. We will be, in a future study of these patients analyse the choroidal architecture during the follow up of our patients.

A study of OCTA in a patient with varicella retinal vasculopathy showed loss of capillary plexus in both SCP and DCP^24^.

OCTA has also been useful in choroidal imaging as described in a case of sympathetic ophthalmia.OCTA of the choroidal vascular revealed flow void pockets initially at inflammatory stage, and this normalized over time into typical granular pattern after initiation of the treatment^25^.

Despite the advantages of being non-invasive and repeatable, OCTA has certain limitations in active retinitis. Its interpretation can be challenging due to projection and motion artifacts and retinal edema due to active retinitis causing an impression of vessel drop out and a loss of the regular “angio-architecture” due to vessel displacements, pruning effects and non-flow areas in edematous areas. The interpretation of OCTA and particularly the FAZ is difficult in patients with gross macular edema and will need longitudinal follow up to assess for enlargement, distortion and possible ischemia.

Other limitations include a relatively small field of view, inability to show leakage, and proclivity for image artifact due to patient eye movement/blinking. Manual segmentation can be tedious and time consuming. The variations in capillary density or vascular thickness are influenced by the type of segmentation. We overcame this limitation by having two observers performing the manual segmentation, comparing the findings and taking the average of the two readings.

## Conclusion

Ours is the largest series of OCTA of retinal vasculature findings in post fever retinitis. Although the presumed etiology was different in our patients, they developed similar changes on OCTA. Quantitative analysis confirmed that the insult was more in the DCP. Serial follow up of these patients will help unravel the vascular changes on the road to recovery.
